# A mixed-methods feasibility study of a comorbidity-adapted exercise program for low back pain in older adults (COMEBACK): a protocol

**DOI:** 10.1186/s40814-022-01097-x

**Published:** 2022-07-02

**Authors:** Katie de Luca, Megan Yanz, Aron Downie, Julie Kendall, Søren T. Skou, Jan Hartvigsen, Simon D. French, Manuela L. Ferreira, Sita M. A. Bierma-Zeinstra

**Affiliations:** 1 Department of Chiropractic, Faculty of Medicine, Health and Human Sciences, Macquarie University, Sydney, Australia; 2 Discipline of Chiropractic, School of Health, Medical and Applied Sciences, CQUniversity, Brisbane, Australia; 3 Discipline of Chiropractic, School of Health and Biomedical Sciences, RMIT University, Melbourne, Australia; 4 Department of Sports Science and Clinical Biomechanics, Centre for Muscle and Joint Health, University of Southern Denmark, Odense, Denmark; 5 Department of Physiotherapy and Occupational Therapy, Næstved-Slagelse-Ringsted Hospital, Slagelse, Denmark; 6 Nordic Institute of Chiropractic and Clinical Biomechanics, Odense, Denmark; 7 Faculty of Medicine and Health, Institute of Bone and Joint Research, The Kolling Institute, The University of Sydney, Sydney, Australia; 8 Department of General Practice and Department of Orthopaedics, University Medical Center Rotterdam, Erasmus MC, Rotterdam, Netherlands

**Keywords:** Back pain, Low back pain, Comorbidity, Multimorbidity, Exercise, Ageing, Older, Feasibility study

## Abstract

**Background:**

The prevalence of low back pain increases with age and has a profound impact on physical and psychosocial health. With increasing age comes increasing comorbidity, and this also has pronounced health consequences. Whilst exercise is beneficial for a range of health conditions, trials of exercise for low back pain management often exclude older adults. It is currently unknown whether an exercise program for older adults with low back pain, tailored for the presence of comorbidities, is acceptable for participants and primary healthcare providers (PHCPs). Therefore, this mixed-methods study will assess the feasibility of an 8-week comorbidity-adapted exercise program for older people with low back pain and comorbid conditions.

**Methods:**

The 3-phased feasibility study will be performed in a primary healthcare setting. PHCPs will be trained to deliver a comorbidity-adapted exercise program for older people with low back pain and comorbidities. Healthcare-seeking adults > 65 will be screened for eligibility over telephone, with a recruitment target of 24 participants. Eligible participants will attend an initial appointment (*diagnostic phase*). During this initial appointment, a research assistant will collect patient demographics, self-reported outcome measurement data, and perform a physical and functional examination to determine contraindications and restrictions to an exercise program. During the *development phase*, PHCPs will adapt the exercise program to the individual and provide patient education. During the *intervention phase*, there will be two supervised exercise sessions per week, over 8 weeks (total of 16 exercise sessions). Each exercise session will be approximately 60 min in duration. A qualitative evaluation after the last exercise program session will explore the feasibility of the exercise program for participants and PHCPs. Progression criteria will determine the suitability for a fully powered randomised controlled trial.

**Discussion:**

This mixed-methods feasibility study will assess an exercise program for older adults with low back pain and comorbidities. Once assessed for feasibility, the exercise program may be tested for effectiveness in a larger, fully powered randomised controlled trial. This information will add to the sparse evidence base on appropriate options for managing back pain in older adults.

**Trial registration:**

Australian and New Zealand Clinical Trials Registry registration number: ACTRN12621000379819p (06/04/2021; https://www.anzctr.org.au/Trial/Registration/TrialReview.aspx?ACTRN=12621000379819p).

**Trial sponsor:**

Macquarie University, Department of Chiropractic, Faculty of Medicine, Health and Human Sciences, Macquarie University, NSW 2109, Australia.

**Supplementary Information:**

The online version contains supplementary material available at 10.1186/s40814-022-01097-x.

## Background

Low back pain is the leading cause of disability worldwide, and the prevalence of low back pain increases over the lifespan with a peak prevalence observed around the ages of 80 to 89 years [1]. Low back pain has a profound impact on the physical and psychosocial health of an older adult. For example, one-fifth of older adults with low back pain cannot care for themselves at home or participate in family and social activities [2]. Additionally, one in every four people aged > 80 years will report moderate to severe low back pain, and people aged > 80 years are three times more likely to have high intensity low back pain than those aged 50–59 years [3]. Whilst numerous national guidelines suggest exercise as a first-line treatment for low back pain [4, 5], older adults with low back pain who visit a general practitioner are 50% less likely to be advised about exercise than younger patients [6].

Comorbid diseases are defined as “any distinct additional entity that has existed or may occur during the clinical course of a patient who has the index disease under study” [7]. Comorbidity may be the effect of medical diseases existing simultaneously, but independently of each other [8], or of diseases that may be related [9]. Research has shown that women with spinal pain were twice as likely to be overweight or obese, have diabetes, and nearly twice as likely to have pulmonary disease and cardiovascular comorbidity, than older women without spinal pain [10]. Furthermore, musculoskeletal conditions such as low back pain are associated with an increased risk of reporting multiple chronic health conditions [11]. Indeed, it is estimated that two-thirds of people aged over 65 years have two or more chronic health conditions [12]. The consequences of comorbidity in older adults include poorer prognosis and increased healthcare use [13]. For instance, with every additional (non-musculoskeletal) comorbidity, there is a 40% increase in the risk of developing persistent low back pain (*OR*: 1.4; 95% *CI*: 1.3 to 1.5) [14]. The widespread exclusion of participants with comorbidities in research is of great concern [15]. There is a need for future trials to evaluate the effectiveness of interventions for older adults who have low back pain and comorbidities.

Exercise has been shown to be beneficial for a wide range of conditions that are associated with ageing such as falls [16], frailty [17], and sarcopaenia [18], as well as comorbidities that co-occur with low back pain [19]. Yet, randomised controlled trials of exercise programs for acute and chronic low back pain often do not include older adults with comorbid conditions [20, 21].

Recently, a comorbidity-adapted exercise intervention was designed and tested for patients with knee osteoarthritis [22], showing that tailored exercise therapy provided significant improvements in pain and physical functioning, and that these improvements were of clinical relevance [23]. It is currently unknown whether an exercise program for older adults with low back pain, tailored for the presence of comorbidities, is acceptable for participants and primary healthcare providers (PHCPs) such as physiotherapists and chiropractors. Therefore, this mixed-methods study will assess the feasibility of a comorbidity-adapted exercise program for older people with back pain and comorbidities. Specific aims directly related to assessing the feasibility are as follows:

1. Determine the ability to recruit eligible older participants and report the retention rate.
2. Evaluate adherence to, and fidelity of, the comorbidity-adapted exercise intervention.
3. Assess the collection and completion of outcome measure data, as well as report adverse events.
4. Evaluate the acceptability of the exercise program to participants and PHCPs.

## Methods

### Design and setting

This protocol describes the design of a mixed-methods feasibility study of a COMorbidity-adapted Exercise program for low BACK pain in older adults (COMEBACK). The study will be conducted in a primary healthcare setting. Chiropractors and physiotherapists (PHCPs) with more than 5 years’ experience in back pain management and rehabilitation will deliver the exercise program. Four PHCPs will be trained in how to deliver the exercise program, via structured, in-person training prior to the development phase of the intervention. The study protocol conforms with the Standard Protocol Items: Recommendations for Interventional Trials (SPIRIT) guidelines [24], with the SPIRIT checklist as Supplementary material 1 Furthermore, as it has been recently shown that exercise interventions for low back pain are poorly reported [25], the intervention has also been reported as per the Template for Intervention Description and Replication (TIDieR) [26] (Supplementary material 2), and the exercise program will comply with the i-CONTENT tool [27]. The study results will be reported according to the CONsolidated Standards Of Reporting Trials (CONSORT) statement extension for pilot and feasibility studies [28]. The trial was registered with the Australian and New Zealand Clinical Trials Registry, reference number: ACTRN12621000379819p (06/04/2021; https://www.anzctr.org.au/Trial/Registration/TrialReview.aspx?ACTRN=12621000379819p. Ethical approval was granted by the Macquarie University Human Research Ethics Committee, reference number: 52021641028032. The trial resources are being funded by a Macquarie University Research Seeding grant; however, they have no role in study design, data collection, and analysis, interpretation of data, or writing of this manuscript.

### Recruitment

The study population is a convenience sample of adults aged 65 years or older who seek care for their low back pain in a primary healthcare setting. Older adults with low back pain will be invited to participate, via in-clinic posters in general practitioner, university, and PHCP clinics. The involvement of general practitioners in the recruitment process of participants in exercise interventions often needs to be supplemented by other recruitment methods [29], so social media posts will also invite community-dwelling older adults with low back pain to participate. Interested older adults will telephone a research assistant, who will screen for eligibility. If patients meet inclusion and exclusion criteria (Table 1), they will be asked to attend an initial appointment. At the appointment, the patient will be provided with a participant information form, and the study is explained verbally by the research assistant. If the patient is willing to participate, written consent will be obtained.

**Table 1:** Inclusion and exclusion criteria

*Inclusion (satisfy all)*	*Exclusion (satisfy any)*
Aged 65 years or older	Patients with radiculopathy, evidence of nerve root compromise, and/or intermittent neurogenic claudication
Non-specific low back pain (within the boundaries of the thoracolumbar junction to gluteal folds) of less than 3-month duration	Patients with medically diagnosed fibromyalgia, a primary chronic pain condition^a^, peripheral neuropathy
Diagnosis of at least one of the target comorbidities: coronary heart disease, hypertension, type 2 diabetes, obesity, chronic obstructive pulmonary disorder, and depression	Diagnosis of serious pathology in the spine (such as fracture, malignancy or metastatic disease, cauda equina syndrome)
Bayliss measure of illness burden score of 3 or above for at least one of the target comorbidities	Physiotherapy or chiropractic treatment for their low back pain in the last 3 months
Sufficient comprehension of the English language to understand exercise instructions	Inability to participate in treatment, e.g. due to transport problems, or unable to mobilise independently
	Patients with an unstable health condition or at risk of a serious adverse event as evaluated by a medical specialist
	Patients with psychosis disorders, posttraumatic stress disorder, obsessive-compulsive disorder, attention-deficit hyperactivity disorder, autism, anorexia nervosa, and bulimia nervosa and patients with an abuse disorder

^a^A primary chronic pain is defined as chronic pain that cannot directly be ascribed to any disease of structural injury[30, 31]

### Participants

In determining the sample size for progression criteria for a pragmatic pilot randomised controlled trial, CTs using a red/amber/green approach, we used the estimation from Lewis et al. [32] with an upper limit red zone (unacceptable) of 45% and a lower limit green zone (acceptable) of 75% for the feasibility outcome protocol adherence (Table 2). The test is a 1-tailed test with suggested alpha (*α*) of 0.05 and beta (β) of 0.1 [32]. Therefore, our target sample size is 24 participants. Participants must have at least one of the following target comorbidities: coronary heart disease, hypertension, type 2 diabetes, obesity, chronic obstructive pulmonary disorder, and depression (diagnosed by a medical specialist). There will be no cost to participants to undertake the exercise program as part of the study.

**Table 2:** The traffic light system for progression criteria of this feasibility study to randomised controlled trial

	Green	Amber	Red
**Trial recruitment**	Recruitment of 24 participants with low back pain and comorbidity within 3 months	Recruitment of 24 participants with low back pain and comorbidity within 3–6 months	Twelve participants with low back pain and comorbidity are *not* recruited within 6 months
	At least 75% retention of participants through follow-up	At least 50% retention of participants through follow-up	Less than 45% retention of participants through follow-up
**Protocol adherence**	At least 75% of participants complete more than half of the exercise program sessions	At least 50% complete more than half of the exercise program sessions	Less than 45% complete more than half of the exercise program sessions
**Outcome data**	At least 80% of participants do not find the outcomes so burdensome that they would not participate in the study again	At least 70% of participants do not find the outcomes so burdensome that they would not participate in the study again	Less than 70% of participants do not find the outcomes so burdensome that they would not participate in the study again
	At least 80% of data is not missing due to attrition or lost to follow-up	At least 50% of data is not missing due to attrition or lost to follow-up	A total of 25% of data is missing due to attrition or lost to follow-up
	Improvements in low back pain and function found clinically relevant by at least 50% of the participants	Improvements in low back pain and function found clinically relevant by at least 25% of the participants	Improvements in low back pain and function found clinically relevant by less than 25% of the participants
	Improvements in quality of life and illness burden by at least 50% of the participants	Improvements in quality of life and illness burden by at least 25% of the participants	Improvements in quality of life and illness burden by less than 25% of the participants
	No serious study-related adverse events during the study period	Less than five serious study-related adverse events during the study period	Five or more serious study-related adverse events during the study period

Green (go), where there are no concerning issues that threaten the success of the trial; amber (amend), where there are remediable issues, thereafter proceeding with caution; red (stop), when there are intractable issues that cannot be remedied

### Comorbidity-adapted exercise intervention

There will be three phases for the comorbidity-adapted exercise program: diagnostic, development, and intervention phases.

#### Diagnostic phase

After written informed consent, the research assistant will collect patient demographics (age, gender, marital status, weight, height, living arrangements, work history, education, highest degree, income, retirement status), medical history, and the participant’s pain history such as duration, trajectory, and symptomatology. A PHCP will then perform a physical and functional examination to determine contraindications and restrictions to an exercise program.

#### Development phase

The first exercise program session, provided by the trained PHCP, will involve a tailored session to adapt the exercise program to the older individual and provide patient education. The exercise program will consist of lower extremity and back extensor muscle-strength training, aerobic training, core stability exercises, balance training, and lower limb and back stretching based on an inventory. The details of the comorbidity-adapted exercise program are presented in Table 3. Adapting the exercise program for individual patients will include tailoring the exercise frequency, intensity, timing, and exercise type based on the presenting comorbid condition(s). Exercise adaptations will be based upon criteria developed from a previously conducted literature search by de Rooji et al. [23] that investigated restrictions, contraindications, and recommended adaptations of the target comorbidities. At this session, the PHCPs will provide patient education, explore the participant’s goals, motivations, and barriers to an exercise program, and provide the participant with paper-based GLA:D Back pain education material [33].

**Table 3:** Description of the comorbidity-adapted exercise program for this feasibility study

Exercise description^a^	Time/dose/repetitions
**Global muscle stretching (whole body)**	**10 min**
**Comorbidity-adapted exercises**	**35 min**
*Muscle strengthening*	
Gluteals	
• Sit to stand	15 repetitions
• Chair squats	10 repetitions
• Standing leg extensions/pulses	10 repetitions/side
• Bridge	10 repetitions/3-s hold
Lumbar erectors	
• Isometric seated back extensions	10 repetitions/3-s hold
• Prone superman (bilateral and alternating unilateral)	5 repetitions/side
Lower extremity	
• Alternating lunges	5 repetitions/side
• Crab walk with resistance tubing	15 repetitions/side
• Standing straight leg abductions (with resistance tubing or isometric)	10 repetitions/side
*Muscle stretching/mobilisation*	
Lower extremity	
• Hamstrings, quadriceps, iliopsoas, gluteals	45 s/side
Lumbar erectors	
• Cat/cow	10 repetitions/2-s hold
• Cobra	45 s
*Core stabilisation*	
• Dead bug	Dependent on patient ability
• Quadruped	Dependent on patient ability
• Seated knee raises with core activation	15 repetitions/side
• Standing/sitting side crunches (weighted)	15 repetitions/side
*Balance training*	
• Standing on towel (progression with eyes closed)	Hold for 15 s each leg (5 repetitions/side)
**Aerobic training**	**15 min**
• Walking on treadmill or cycling	Dependent on patient ability/10 min
• Step ups	5 min

^a^Adapting the exercise program will include tailoring the exercise frequency, intensity, timing, and exercise type based on the presenting comorbid condition(s)

#### Intervention phase

The exercise program will consist of 16 exercise sessions over an 8-week program. There will be two supervised sessions per week with each session being approximately 60 min in duration. Sessions will be offered onsite at the Macquarie University, in a designated group exercise space. PHCPs will deliver multiple exercise sessions to participants within the program. The Borg Rate of Perceived Exertion scale will be used to monitor training intensity during each supervised exercise program session. Educational and coaching strategies will supplement the comorbidity-adapted exercise program throughout each supervised session [34], with adaptation to the exercise program patient directed.

### Data collection

Data will be collected in person by the research assistant at baseline, 4 weeks, and at 8 weeks after the first exercise program session. Patient demographics and outcome measurement data will be collected in the REDCap — Research Electronic Data Capture system and exported to an Excel spreadsheet. De-identified data will then be imported to STATA/IC® (StataCorp, Texas) for analysis. Qualitative interviews will be performed at 8 weeks, and interview data will be stored securely and transcribed verbatim.

### Feasibility measures

Predetermined progression criteria are the primary outcomes for this study. A traffic light system of green (go), amber (amend), and red (stop) will be used to determine progression of this feasibility study to a fully powered randomised controlled trial (Table 2) [35]. The study will not be feasible to progress if there are intractable issues that cannot be remedied [35].

#### Patient recruitment, retention, and intervention adherence

This study will determine how long it takes to recruit 24 participants, report how many older adults are screened, and how many meet eligibility criteria. Attendance will be recorded at each exercise session, by the PHCP. If a participant fails to attend an exercise session, the research assistant will contact the participant to record a reason for not attending the session and discuss additional support to attend future sessions to prevent dropout. Study retention rates will report the percentage of participants who complete the 8-week exercise program and provide complete data through follow-up. Data on adherence will be collected using a smartphone application. On completion of each exercise session, participants will scan a unique RedCap-generated QR code that will open a brief survey that will document the completion of the exercise session, completion of each component of the exercise session, and provide feedback on their exercise session adherence.

#### Barriers and facilitators for clinicians

One-on-one, semi-structured interviews will be held with PHCPs at the end of the exercise program to discuss the barriers and facilitators of delivering the exercise program.

### Clinical measures

Clinical outcome measures and exercise program-specific outcomes will be collected at baseline (*diagnostic phase*), 4 weeks, and 8 weeks (post treatment) (Table 4). Outcome measures will include a physical and functional assessment performed by the PHCP and a self-reported questionnaire completed by the participants as suggested by the core outcome sets for low back pain [36] and multimorbidity [37].

**Table 4:** Schedule of quantitative data to be collected at baseline and follow-up intervals

	Baseline	4 weeks	8 weeks
Patient demographics	x		x
Physical and functional assessments	x	x	x
Low back pain severity	x	x	x
Oswestry Disability Index	x	x	x
EQ-5D-5L	x	x	x
Clinical frailty scale	x	x	x
Bayliss measure of illness burden	x	x	x
Hospital Anxiety and Depression score	x	x	x
Sit-to-stand test	x	x	x
6-min walk test	x	x	x
Global perceived effect		x	x
Participant satisfaction with treatment		x	x
Adverse events		x	x

#### Physical and functional assessment

The participant will perform the standing forward bending test, back extensor endurance test, and the trunk flexor endurance test. The sit-to-stand test will be performed to assess the functional capacity of participants [38]. Participants will be asked to sit in a chair and stand five times without the use of their arms as quickly as possible. This test will then be repeated, and an average test time will be calculated [39]. The 6-min walk test will be carried out by patients on a 30-m walkway to assess aerobic capacity and endurance. The distance covered over a time of 6 min is used as the outcome by which to compare changes in performance capacity. Patients will be instructed to walk their maximum distance in a 6-min period of time. The total distance covered in metres during 6 min of walking will be scored [6].

#### Self-reported outcome measures

Low back pain severity will be assessed using an 11-point numerical rating scale (NRS) ranging from 0 (no pain) to 10 (worst pain imaginable). Two severity questions will be asked at baseline, including current pain and average pain over the last week. Low back pain-related disability will be assessed using the Oswestry Disability Index (ODI). The ODI consists of 10 items representing different health constructs. The total score of the ODI is calculated by adding all scores of applicable items, dividing the obtained score by the maximal total score, and by multiplying the result by 100 to obtain a percentage score [40].

##### Health-related quality of life

The health-related quality of life will be measured using the EQ-5D-5L instrument. EQ-5D-5L is a generic instrument comprised of five questions about mobility, self-care, usual activities, pain and discomfort, and anxiety and depression, each measured on a 5-point scale from no problems to extreme problems. Australian EQ-5D-5L instrument scores are consistent with EQ-5D-5L utility and visual analog scores reported for other countries [41].

##### Illness burden

The Bayliss measure of illness burden is used to indicate how much illness affects the participant’s daily life and is measured using a 5-point scale for each individual condition and summed to a total score across conditions [42].

##### Mental health

The Hospital Anxiety and Depression score (HADS) will be used to measure anxiety and depression and has been validated across the lifespan [43]. It consists of 14 items, with scores that range between 0 and 21: scores from 0 to 7 are ‘normal’, from 8 to 10 ‘mild’, from 11 to 15 considered ‘moderate’, and from 16 to 21 ‘severe’ [44, 45].

##### Frailty

The Clinical Frailty Scale will be used to summarise the overall level of fitness or frailty of an older adult [46]. The scale is a way to summarise information from a clinical encounter with an older person, which is useful to screen for and quantify an individual’s overall health status [46].

### Exercise program-specific outcomes

At 4 weeks and 8 weeks, global perceived effect and patient satisfaction data will be collected. Patients will be asked to rate perceived effectiveness on a scale of 1–9, with a score of 1 meaning much better, 5 meaning no change, and 9 meaning much worse [47]. Participant satisfaction with treatment will be measured using the numerical rating scale (0–10), with higher scores indicating greater satisfaction [48].

### Comorbid conditions and adverse events

PHCPs and the research assistant will not monitor comorbid conditions regularly at exercise program sessions. The patient will continue their normal contact with their general practitioner and other healthcare professionals.

#### Adverse events

Adverse events are defined as any undesirable experience occurring during the study (regardless of whether this was related to the treatment) by the participant [49] and will be reported by both the participant and PHCP. Classification of adverse event severity is shown in Table 5. The PHCP will have a current first aid certificate and will record any adverse events that happen during the feasibility study period (8 weeks). Adverse events or discomfort experienced by participants during this study may relate to possible side effects from performing the exercise program. Participation in the exercise program may exacerbate individual comorbid condition(s), and some participants may also be at risk of falling. During the administration of the exercise program, should a severe exacerbation of a comorbid condition arise, an ambulance will be called, and the event will be documented in the patient’s file. There is also the possibility of uncovering an unexpected medical condition during the diagnostic phase of the exercise program. Should this arise, the participant will be referred to their general practitioner by the PHCP with a letter stating the relevant findings. In both cases, continuation in the study will be at the discretion of the participant and/or the participant’s general practitioner.

**Table 5:** Classification of adverse event severity

*Mild*: asymptomatic or mild symptom, requiring self-care only (e.g. ice/heat, over-the-counter analgesic)	
*Moderate*: limiting age-appropriate activities of daily living (e.g. work, school) OR sought care from a medical doctor	
*Serious*: results in death OR is a life-threatening AE OR an AE resulting in inpatient hospitalization or prolongation of existing hospitalization for more than 24 h, a persistent or significant incapacity or substantial disruption of the ability to conduct normal life functions, a congenital anomaly/birth defect	

Changes in low back pain will be reported by participants via a daily SMS asking for a numerical ratings scale score (0–10) on the severity of their low back pain throughout the 8-week exercise program. If SMS data identifies an increase in low back pain, participants will be contacted to be asked whether they felt the exercise program may have caused an increase in low back pain. Additionally, the daily SMS will ask about any undesirable experiences related to the exercise program. If the SMS identifies an exacerbation of a comorbidity, the participant will be contacted to be asked about the nature of the adverse event.

### Qualitative evaluation

After their last session, participants and PHCPs will be interviewed by a researcher with relevant qualitative expertise to explore the feasibility of the exercise program and barriers and facilitators to their participation.

#### Participant interviews

At the end of the study period, participants will be invited to take part in a one-on-one semi-structured interview to discuss the acceptability of the exercise program, fidelity of delivery, and the barriers and facilitators to adherence with the exercise program. Arrangements for the interview will be made at the time of the last exercise program session (8 weeks), with interviews carried out by a researcher who did not work with the delivery of the exercise program.

#### PHCP interviews

At the end of the exercise program, PHCPs responsible for administering the exercise program will be invited to take part in a one-on-one semi-structured interview to determine the degree to which the exercise program was delivered as intended (fidelity). They will also be asked to discuss the barriers and facilitators of delivering the exercise program to older adults with low back pain and comorbidities. The interviews will be carried out by a researcher who did not work with the delivery of the exercise program.

### Analysis

The analysis will include a descriptive analysis and qualitative evaluation. Outcome measures change scores will be calculated by subtracting the postexercise program scores (8 weeks) from baseline scores and means with standard deviation (SD) and 95% confidence intervals (CI) reported. All one-on-one semi-structured interviews will be electronically recorded, transcribed, and imported into the NVivo data management program (NVivo qualitative data analysis software; QSR International Pty Ltd.). Transcripts will be coded to the domains of the Theoretical Domains Framework to extract the acceptability, barriers, and facilitators of the exercise program [50]. Transcripts will be coded systematically and iteratively until saturation is achieved. PHCP diaries will be reviewed to identify barriers to inform future trials.

### Participant withdrawal

Participation is voluntary, and withdrawal from the study at any stage is at the discretion of the participant, and relationships with the study’s researchers, PHCPs, or Macquarie University will not be affected. The research assistant will notify the chief investigator if a participant wishes to withdraw from the trial, and the participant will be asked to complete and sign a withdrawal form. Providing reasoning for withdrawal is voluntary and will be documented. Participants will still be provided with their usual care from their general practitioner if they wish to withdraw from the study.

### Data protection, storage, and dissemination

Participants will be provided with a unique identification code upon providing written informed consent. Quantitative data will be collected in REDCap, exported to an Excel spreadsheet, and then de-identified data will be imported to STATA/IC® (StataCorp, Texas) for analysis. The master dataset in Excel will be stored in a secure server, and access to the final dataset will be granted for the research team as required. Participant confidentiality will be maintained, and data will be non-identifiable in conference presentations and publications disseminating the study results. The full protocol will be made available via peer-review publication, and participant-level dataset will be made available via contacting the corresponding author.

## Discussion

This protocol describes the rationale, design, and assessment for a comorbidity-adapted exercise program for older adults with low back pain. Although exercise is a recommended first-line treatment for low back pain, older adults are 50% less likely to be advised about exercise than younger patients [6]. Qualitative studies have revealed that a lack of professional guidance, inadequate distribution of information, and limited participation in physical activity programs are barriers to the uptake of exercise programs in older adults [51]. Similar to younger adults, motivation to engage in an exercise program is often a problem in older adults, which may be overcome by appropriate access to affordable, convenient, and stimulating exercise programs. Our study will use a qualitative evaluation to better understand barriers and facilitators to the uptake of exercise programs in older adults with low back pain. Importantly, in this study, the exercise program is developed to overcome physical limitations due to ageing and comorbidity. Older adults are twice as likely to have comorbidity if they have spinal pain [10] and often have pronounced health consequences due to comorbidity [52]; thus, the exercise program has been designed to be physiologically appropriate for older adults with low back pain and comorbidities. That is, that the exercise program is designed to be safe, acceptable, and will address the main impairments and functional limitations of older people. Other studies implementing standard exercise programs designed for younger adults with low back pain for older adults do not account for comorbidity that is present.

Results from this feasibility study will inform the design of an intervention for a large community-based randomised controlled trial. The fully powered trial will aim to investigate the long-term effects of an individualised, physiologically appropriate, exercise program on older people’s capacity to self-manage their low back pain, on low back-related disability, and the effect on specific comorbid conditions. If an exercise program appropriate for older adults with low back pain and comorbidities is found to be effective, further research would be needed to implement the exercise program in both primary care and community settings. This may result in improved health service delivery for older adults with low back pain and decreased healthcare expenditure in this population. With low back pain being the greatest cause of disability globally, and the prevalence of comorbid conditions increasing due to an ageing population whereby older adults have a higher number of comorbid conditions, research focused on older people with spinal pain and comorbidities is urgently needed.

## Supplementary Information

**Additional file 1: Supplementary material 1**. SPIRIT 2013 Checklist: Recommended items to address in a clinical trial protocol and related documents.

**Additional file 1: Supplementary material 2.** Template for Intervention Description and Replication (TIDieR).

## Data Availability

Not applicable.

## Abbreviations

AE: Adverse events
CI: Confidence Interval
CONSORT: Consolidated Standards of Reporting Trials
HADS: Hospital Anxiety and Depression score
NRS: Numerical ratings scale
OR: Odds ratio
ODI: Oswestry Disability Index
PHCPs: Primary healthcare providers
REDCap: Research Electronic Data Capture
RCTs: Randomized controlled trials
SPIRIT: Standard Protocol Items: Recommendations for Interventional Trials
TIDieR: Template for Intervention Description and Replication
