# Supplementary material for: A mixed-methods feasibility study of a comorbidity-adapted exercise program for low back pain in older adults (COMEBACK): a protocol
Source: Pilot Feasibility Stud. 2022 Jul 2;8:133. doi: 10.1186/s40814-022-01097-x (PMC9250189; doi:10.1186/s40814-022-01097-x)
Supplement: Supplementary file 2 — Additional file 1: Supplementary material 2. Template for Intervention Description and Replication (TIDieR). [file 40814_2022_1097_MOESM2_ESM.pdf]

TIDieR

COME-BACK (COMorbidity-adapted Exercise program for low BACK pain in older adults)

**Why:**	It is currently unknown whether an exercise program for older adults with low back pain, tailored for the presence of comorbidities, is acceptable for participants and primary healthcare providers. Therefore, this mixed methods study will assess the feasibility of a comorbidity-adapted exercise program for older people with back pain and comorbidities.
**What (material):**	In the first exercise program session a trained PHCP will provide the participant with paper-based, GLA:D Back pain education material.
**What (procedures):**	There will be three phases: diagnostic, development and intervention phases. During the diagnostic phase, a physical and functional examination will be performed to determine contraindications and restrictions to exercise. During the development phase, the first exercise session is a tailored to adapt the program to the individual and provide patient education. During the intervention phase, the exercise program includes lower extremity and back extensor muscle-strength training, aerobic training, core stability exercises, balance training, and lower limb and back stretching.
**Who provided:**	Chiropractors and physiotherapists (primary healthcare providers (PHCPs)) with more than five years' experience in back pain management and rehabilitation will deliver the exercise program. PHCPs will be trained in the exercise program and to provide the program in accordance with the protocol.
**How (mode of delivery; individual or group):**	Sessions will be offered, face to face, in a designated group exercise space, at Macquarie University.
**Where:**	Sessions will be offered, face to face, in a designated group exercise space, at Macquarie University.
**When and how much:**	The exercise program will consist of 16 sessions throughout the eight-week exercise program. There will be two supervised sessions per week, with each session being approximately 60 minutes in duration. The Borg Rate of Perceived Exertion scale will be used to monitor training intensity during each supervised exercise program session.
**Tailoring:**	Yes, this intervention is planned to be individualised. Adapting the exercise program for individual patients will include tailoring the exercise frequency, intensity, timing, and exercise type based on the presenting comorbid condition(s).
**How well (planned):**	At the end of the study period, participants and PCHPs will take part in a one-on-one semi-structured interview to determine the degree to which the exercise program was delivered as intended (fidelity).
